# Recent Developments in the Methods of Estimating Shooting Distance

**DOI:** 10.1100/tsw.2002.140

**Published:** 2002-03-07

**Authors:** Arie Zeichner, Baruch Glattstein

**Affiliations:** Division of Identification and Forensic Science, Israel Police National Headquarters, Jerusalem 91906, Israel

**Keywords:** shooting distance, firing distance, gunshot residue, GSR, Griess test, MGT

## Abstract

A review of developments during the past 10 years in the methods of estimating shooting distance is provided. This review discusses the examination of clothing targets, cadavers, and exhibits that cannot be processed in the laboratory. The methods include visual/microscopic examinations, color tests, and instrumental analysis of the gunshot residue deposits around the bullet entrance holes. The review does not cover shooting distance estimation from shotguns that fired pellet loads.

## INTRODUCTION

The range from which a weapon has been fired is an important component in the reconstruction of firearm-related offenses (murder, suicide, and accident). The firing distance estimation is based on the examination of the appearance of the bullet entrance hole and the examination of gunshot residue (GSR) patterns around the hole using various techniques. Although many authors in the field use the phrase "shooting distance determination", we prefer to use the term "estimation" instead of "determination" because of the intrinsic inaccuracy of the examination. The reason for this is a high variability of the GSR patterns from shot to shot when using the same weapon and ammunition. By GSR, we mean all the materials emitting from the muzzle during shooting and accompanying the projectile. These include gunpowder and primer residues as well as metal particles from the bullet and the cartridge case. In most of the shooting cases in which there is a need for a firing distance estimation, the victim or the victim's clothing has to be examined. In many cases, bullets hit surfaces of various parts of the human body directly without passage through any intermediate medium. In some instances, other exhibits that happened to be targets of shooting have to be examined. Such exhibits may be cars, walls, doors, windows, furniture, etc. Many of them cannot be processed in the laboratory.

In this review, we will report on the recent developments (in about the last 10 years, since the two comprehensive treatises on the subject were published[1,2]) in the methods of visual/microscopic, color test, and instrumental analysis of the entrance bullet holes and GSR patterns around them for shooting distance estimation. A comprehensive list of references on advances made in the field over the last 3 years can be found elsewhere[3]. We will not deal here with shooting distance estimation for targets shot by pellet loads from shotguns. In our view, there were no significant developments recently in this area, which is described comprehensively elsewhere[1,4,5].

## VISUAL AND COLOR TESTS

### Clothing Targets

GSR patterns around the entrance bullet holes consist of propellant residues, metallic residues from bullets (e.g., lead and copper), as well as primer residues. These residues can be detected visually/microscopically if the target cloth is of a light enough color. However, in most of the cases, there is a need for color chemical tests or instrumental analysis to assess the GSR patterns around the entrance bullet holes.

In a series of three articles, Dillon reports[6,7,8] on the modified Griess test (MGT) as a color test for nitrites and recommends a protocol for GSR examinations in muzzle-to-target-distance estimations. In the original procedure reported by Walker[9], Griess reaction is used. In this test, Griess reagent consisting of sulphanilic acid and α-naphthyl amine in acetic acid aqueous solution is used. The detection of free nitrite ions is based on the formation of diazonium ion from sulphanilic acid and nitrite. The diazonium ion couples with α-naphthyl amine to form an orange azo dye. In the modified test, Dillon proposes to use α-naphthol instead of α-naphthyl amine (the Walker test) or N-(1-naphthyl)-ethylenediamine dihydrochloride. According to him, both of the replaced reagents are carcinogenic. However, in the literature on the chemical safety data[10], N-(1-naphthyl)-ethylenediamine dihydrochloride is not reported as a carcinogen. In fact, we are using this reagent with sulphanilamide routinely for “our” MGT [11,12,13,14]. MGT does not refer to one specific, defined test. In fact, it appears that every author who introduces any modification to the original Griess test calls it MGT. The proposed protocol[8] includes visual, microscopic, and chemical (lead and nitrites) tests. It recommends first conducting the MGT and then the sodium rhodizonate test for lead. The reason for this sequence is because the rhodizonate is applied directly on the target. Dillon contends that particulate lead is a random nonreproducible phenomenon, whereas the presence of vaporous lead is quite significant in that it is found principally at wounds from closer ranges.

Glattstein et al. reported on an improved method of determining shooting distance estimation from clothing[12]. The novel part of the method includes transferring total nitrite (nitrite ions and unburned smokeless powder residues) from the target to an adhesive lifter. After the transfer, lead and copper deposits around the bullet entrance hole are partially extracted consecutively to the Benchkote (Whatman) filter papers moistened with diluted acetic acid and ammonia solutions, respectively. Their patterns are visualized by rhodizonate (lead) and rubeanic acid (copper). The MGT is carried out after alkaline hydrolysis of the smokeless powder residues on the adhesive lifter. The purpose of lifting gunpowder residues from the shot cloth target is to eliminate interference caused by conducting MGT directly on the target, with or without the hydrolysis step. It was found that almost a complete transfer of gunpowder residues to the adhesive lifter was obtained, and the vaporous lead and copper are not transferred to the adhesive lifter. The widely used MGT detects only free nitrite ions formed from the combustion of smokeless powder. The unburned smokeless powder particles cannot be detected by this method. Alkaline hydrolysis prior to the MGT has been proposed to increase the sensitivity of the test for gunpowder residues[15]. The purpose of the alkaline hydrolysis is to cause disproportionation of the unburned nitrocellulose and nitroglycerine (the main components of the smokeless powder) to

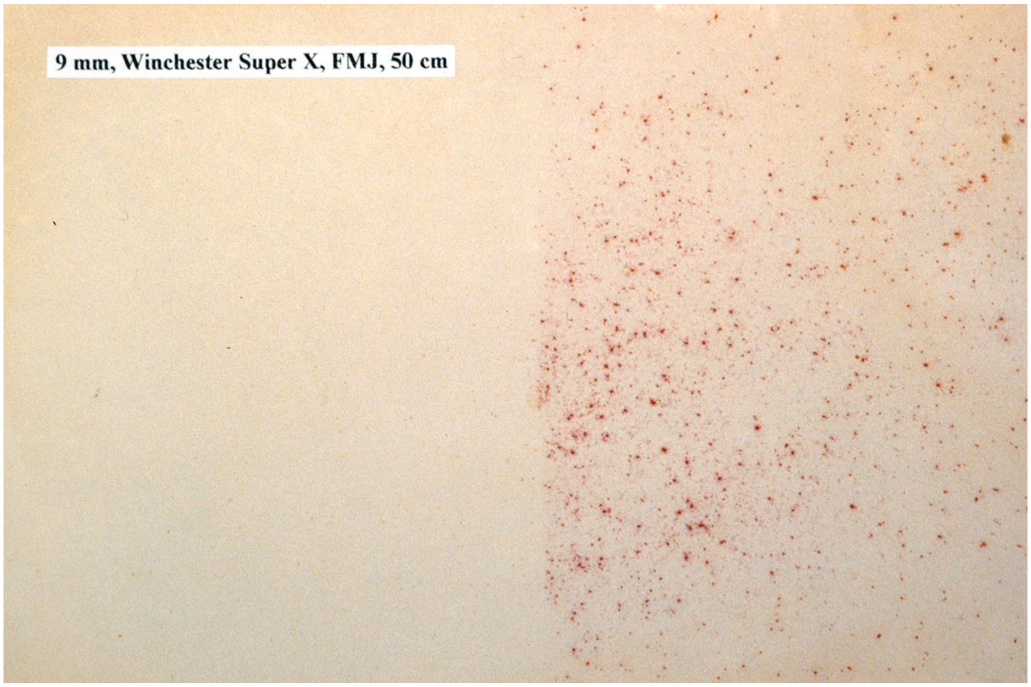

**FIGURE 1**. A test shot on a white cotton cloth; ammunition 9-mm Winchester super-X FMJ, distance 50 cm. A color photograph of the target after application of MGT: (left) without the hydrolysis prior to MGT, (right) with the hydrolysis prior to MGT.

carbonyl compounds and free nitrite ion, thus increasing the available amount of nitrite ions for MGT. The importance of the hydrolysis step in the gunpowder residue visualization is demonstrated in Fig. 1. As can be seen in this type of ammunition (Winchester Super-X), the difference in patterns obtained with and without hydrolysis is very great. Thus, in such cases, it is very important to carry out this step prior to the MGT. However, not all ammunition types demonstrate such a difference. Before starting the estimation of the shooting distance, it is desirable to determine that the hole is a bullet entrance hole. This can be done by applying methods for chemical visualization of lead (rhodizonate) and copper (rubeanic acid) at the perimeter of the hole. However, it was observed that the color tests do not give positive results in all shooting cases, although it was known that a lead bullet or a full metal jacket (FMJ, brass) bullet was used.

In recent years, several ammunition companies have introduced lead-free ammunition. This technology uses lead-free primers and totally metal-jacketed (TMJ) bullets. The lead bullet core is encased in a metal alloy jacket, thus no lead is exposed at the base where hot gases can vaporize lead (as in conventional full jacketed or lead bullets). In such cases, because lead is not a component of the GSRs, lead patterns cannot be detected for firing distance determination. The Zincon reagent that gives a blue-colored complex with elements zinc and titanium was proposed for firing distance estimation in the case of lead-free ammunitions that have zinc and titanium in the primer[16]. As a side reaction, copper, which among other metals comes from the projectiles or cartridge cases, also forms a blue-colored complex with the reagent.

A modified sheet-printing method for the detection of lead patterns was reported by Stahling[17]. Instead of using cellulose hydrate foil, a plastic-based photographic paper was used as a substrate for transfer of metallic gunshot elements from cloth.

Alakija et al.[18] studied the damage to various cotton clothing targets from different firearms as a function of shooting distance. They found, for example, that .22-caliber pistols always produced “stellate” (“cruciform”) tears at tight contact and loose contact ranges; nonstellate defects were produced by this pistol at ranges of 2 cm or greater. Stellate defects were not produced by any studied firearm at ranges greater than 8 cm.

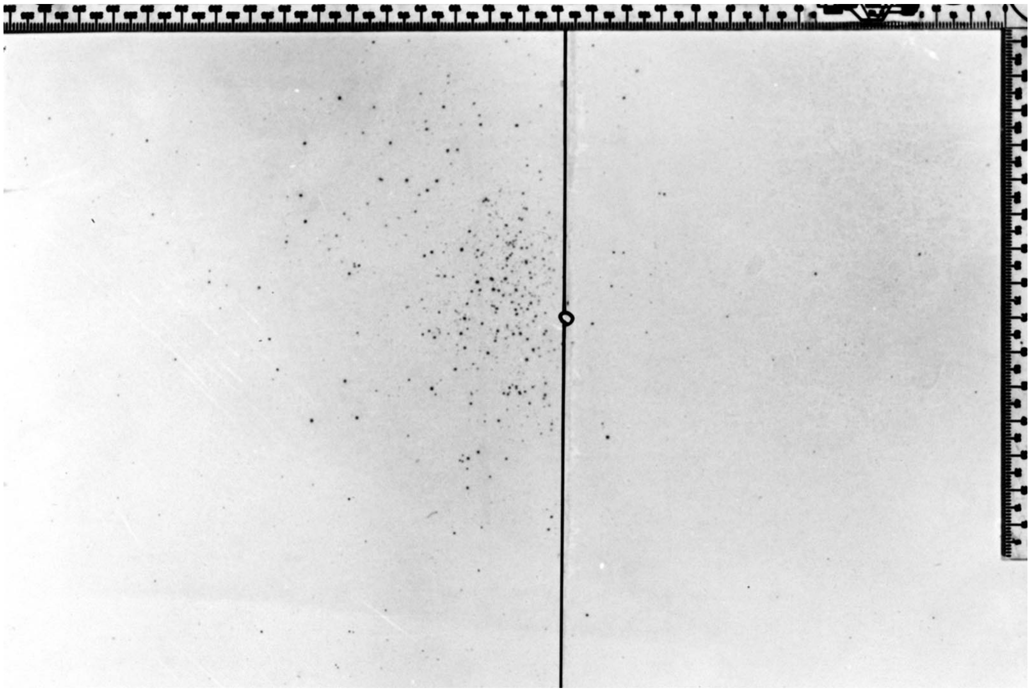

**FIGURE 2**. The effect of machine washing on the total nitrite visualization on a white cotton cloth; ammunition 9-mm parabellum GFL FMJ, 25-cm shooting distance. Black and white photograph of the target: (left) the target without washing, (right) after washing.

## Persistence of GSR on Clothing

Several studies dealt with possible effects of various factors on clothing items after shooting with regards to the shooting distance estimation[8,19,20,21,22,23]. Most of these found that mechanical handling of clothing or soaking them in blood, still water, or running water considerably decreases the amount of GSR around the bullet entrance holes. Thus Emonet et al.[22] report that the medical manipulations of clothing lead to an increase of the loss of visible and nitrated GSR of about 30 to 40%. Even et al.[19], on the other hand, did not find a significant effect of soaking in still water on the obtained GSR patterns. Also L. Haag[20] found that a static extraction procedure utilizing a 12- to 24-h immersion in an aqueous blood-removal solution does not alter significantly the GSR patterns on the shot cloth targets. In casework, sometimes requests are received to estimate shooting distance from clothing items that underwent machine washing. Vinokurov et al.[23] conducted a study to assess the effect of machine washing or brushing of clothing items on GSR patterns (gunpowder residues and lead and copper deposits) around bullet entrance holes. Results show that those treatments decrease considerably (machine washing more than brushing) the amount and density of GSR. However, for close shooting distances, not all of the GSR deposits are removed. Remaining patterns can be visualized by specific color reactions and used for shooting distance estimation. Figs. 2 and 3 illustrate some of the results. This study shows that the absence of GSR patterns around the bullet entrance hole is a clear indication that shooting was not at close range.

## Exhibits that Cannot be Processed in the Laboratory

In our experience, casework mostly involves examining exhibits for shooting distance estimation that are the victims' clothing. Sometimes, however, we are asked to determine shooting distance

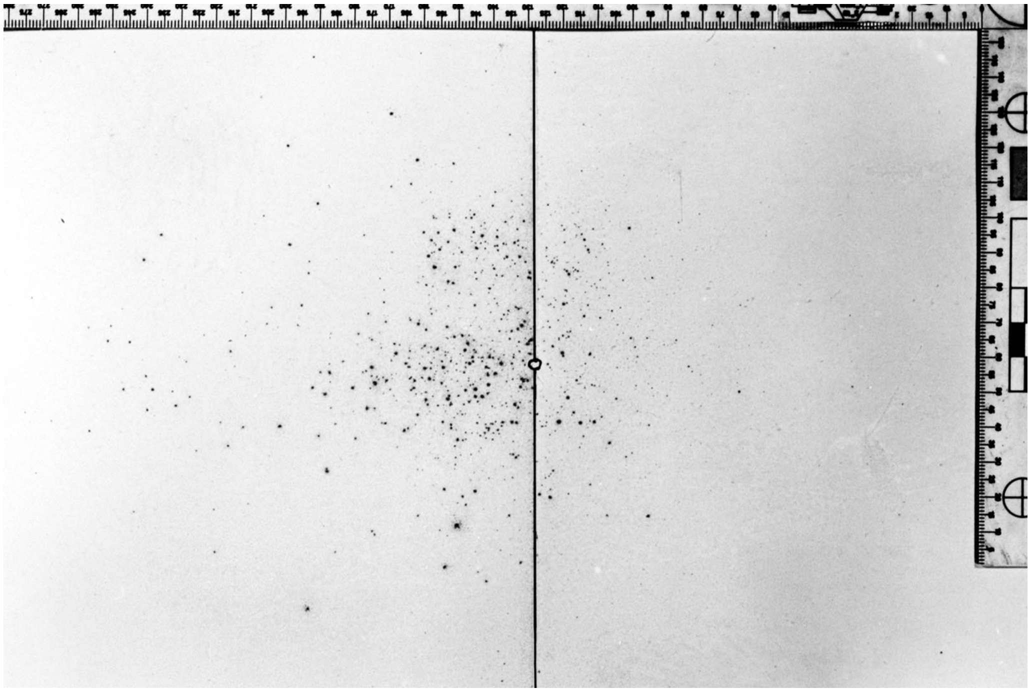

**FIGURE 3**. The effect of brushing on the total nitrite visualization on a white polyester cloth. Same ammunition and distance as in Fig. 2. Black and white photograph of the target: (left) the target without brushing.

from exhibits that cannot be processed in the laboratory, such as cars, walls, doors, windows, or furniture (made of wood, plastic, leather or fabric).

Glattstein et al.[13] examined the feasibility of the method developed for clothing[12] (described above) for additional materials: galvanized steel, glass, plywood, and high-pressure laminated plastic sheets of melamine and phenolic materials (Formica). It was found for all tested target materials and shooting distances that the amounts and densities of the discharge residues detected visually (without any treatment) were considerably smaller than those obtained after chemical treatments. This effect was particularly pronounced in the case of glass, where blackening could hardly be observed even from contact shooting ranges. Total nitrite patterns visualized on the lifters applied on the various targets were similar to those obtained on the lifters from the cotton cloth at relatively short shooting distances, i.e., up to about 25 cm. The patterns were similar in terms of the amount of particles and their density around the entrance bullet hole. Fig. 4 demonstrates this result on plywood and on glass. As shooting distances become greater, the number and density of nitrite spots on the lifters from all the tested materials targets decrease considerably in comparison to the lifters from the cotton cloth for the same distance, the plywood target being the most similar. Based on the obtained results in this study, the recommended method for determining shooting distance estimation from the objects that cannot be processed in the laboratory is as follows:

1. Application of the adhesive lifter to the target.
2. Visualization of the lead deposits on the target by the rhodizonate test. It is recommended to photograph the lead pattern because of the instability of the color.
3. Visualization of the copper deposits on the target by the rubeanic test.
4. Visualization of the total nitrite on the lifter in the laboratory.

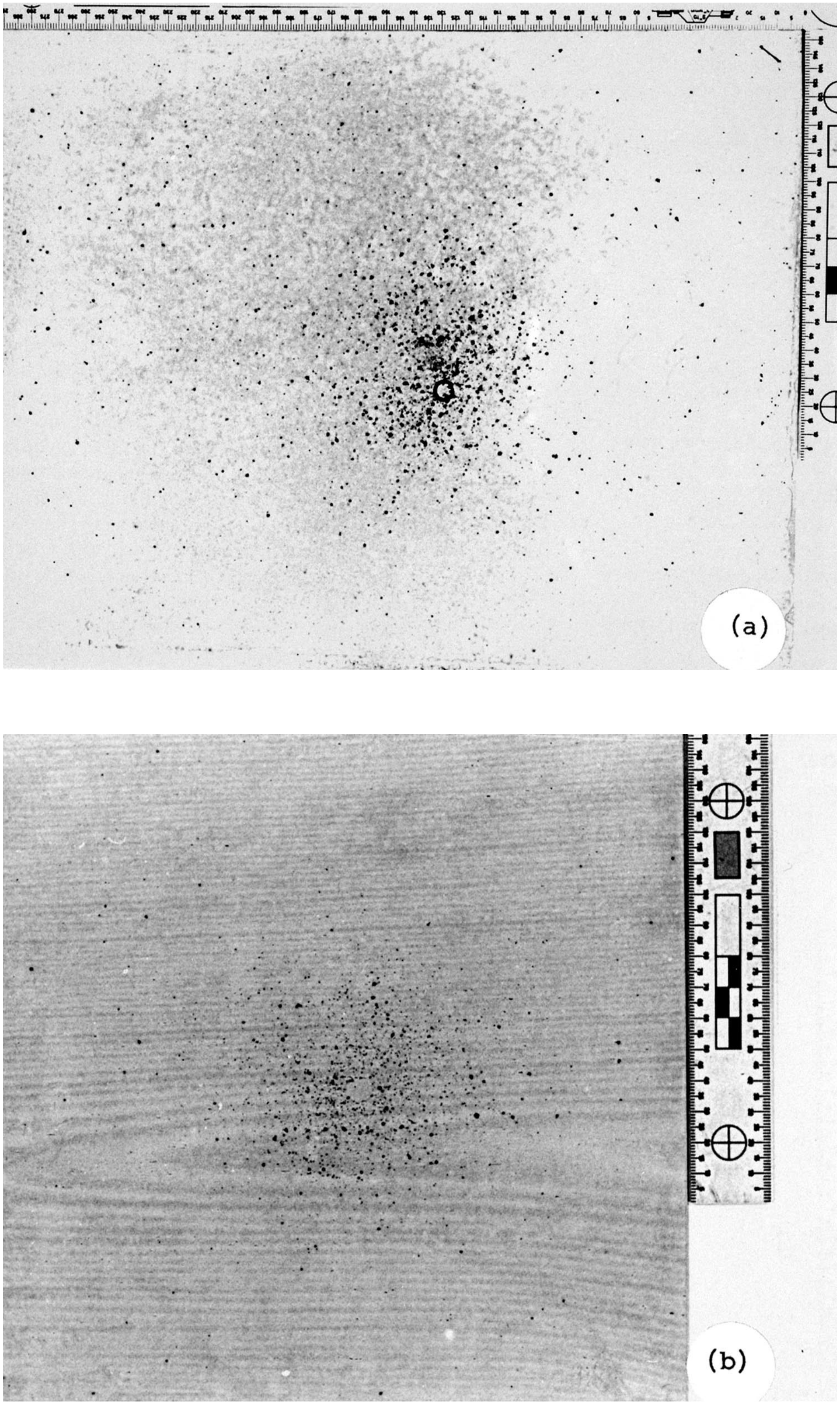

**FIGURE 4**. Black and white photograph of the visualized total nitrite patterns on the adhesive lifters applied to various target materials after shooting (ammunition 9-mm parabellum FMJ, distance 25 cm). (a) White cotton cloth. (b) Plywood. (c) Glass.

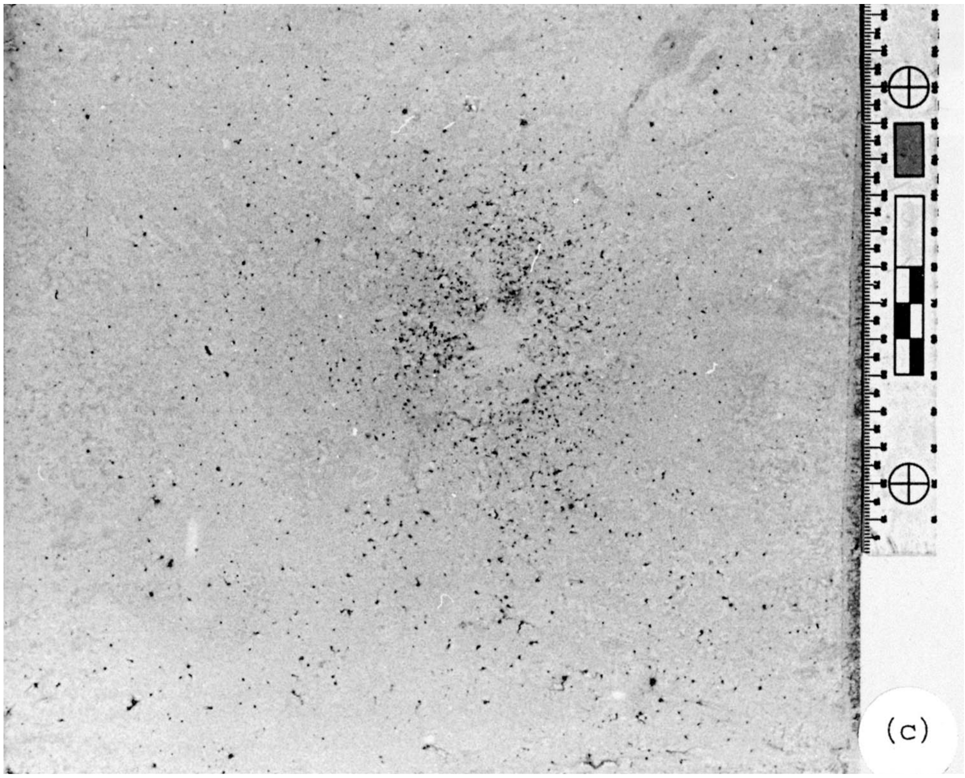

**FIGURE 4c.**

To improve the accuracy of the shooting distance estimation, test firings should be carried out using target materials as similar as possible to the materials of the examined evidence. If there is no possibility of conducting test firing at a material similar to the evidence, then test firings should be carried out on cotton cloth. In such a case, the visualized pattern of the total nitrite will be sufficient to demonstrate that the shooting distance on the evidence was equal to or below the shooting distance at which similar visualized patterns of the total nitrite are obtained on the cotton cloth.

## Human Body as a Target

In many shooting cases, bullets hit surfaces of various parts of the human body (mostly the head) directly, without passage through any intermediate medium. For the purpose of assessing the shooting distance, most of the forensic literature describes only visual/microscopic methods for examination of the appearance of the wound and discharge particle patterns around it[1,4,24].

Shooting distances on human body surfaces can be divided roughly into four ranges: contact, near contact range, intermediate range, and distant range[1,4]. In *contact* wounds, the muzzle of the weapon is held against the surface of the body at the time of discharge. The appearance of tearing, scorching, soot, or the imprint of the muzzle characterizes contact wounds. In *near contact* wounds, the muzzle of the weapon is not in contact with the skin, being held a short distance away (a few centimeters). At near contact range, a wide zone of powder soot overlaying seared blackened skin surrounds the entrance wound. An *intermediate range* gunshot wound is one in which the muzzle of the weapon is held away from the body at the time of discharge, yet is sufficiently close so that gunpowder grains expelled from the muzzle along with the bullet produce "powder tattooing" of the skin. Microscopic examination is conducted to verify the presence of partially burned or unburned gunpowder particles. In the *distant range*, no damage effects or discharge particle patterns are observed around the gunshot wound.

Although sodium rhodizonate and rubeanic acid reagents were proposed for the visualization of lead and copper patterns around the gunshot wounds[25,26], in practice, the authors are not aware of any chemical tests that are conducted for the estimation of shooting distance on cadavers.

As in cases of clothing and other objects, many problems can be encountered when the assessment of shooting distance on cadavers is based merely on visual and microscopic examinations. The typical problems are[1,4,24]:

1. When small-caliber ammunition (short .22 or .32 Smith & Wesson) is used, the typical features of contact gunshot wounds may be absent.
2. Gunpowder tattooing may not be produced on the skin in hairy parts of a body, and gunpowder particles will be hardly discernible.
3. Whenever shots were inflicted through glass panes, glass particles may produce visual patterns that can be similar to the gunpowder tattooing around a gunshot wound[3,6].
4. The discoloration characteristic of a decomposed body can be similar to the color of soot. Furthermore, it may also mask tattoo marks.
5. In rare instances, insects may produce patterns that resemble gunpowder tattooing on cadavers.

An additional unique problem pertaining only to the human body is that there is no possibility of conducting test firing on the same material as in the case of other exhibits. The proposed solution for test firings was to use various simulant materials. Recommendations for those materials were based on the studies comparing those materials to the skin of some animals, like rabbits or pigs[27]. The conclusions of this work were that blotter paper is an acceptable skin simulant at ranges less than 18 in. Beyond this distance, the patterns on the blotter paper were smaller than those on the rabbits, meaning that the blotter paper was less sensitive. In a quite exceptional study, M. Haag and Wolberg conducted experiments on various simulant materials in comparison to live human skin[28]. The comparison was based on various visual and microscopic characteristics (without chemical treatment) of the GSR on the targets. A specially designed experimental setup made it possible to expose part of an arm to the residues. Kevlar vests were laid over each side of the arm to protect the remaining portions of the arm from the residues, allowing for additional shots on fresh skin. The study was carried out at ranges of 2 to 4 ft. They found that the simulants that most closely represent the human skin are fresh pig skin, twill jean cloth, Whatman #1 blotter paper, and Whatman #10 Benchkote. Fresh pig skin was the most accurate overall simulant tested, while the twill jean and blotter paper were more accurate from 2 to 3 ft, and the Benchkote was more accurate at 3 to 4 ft.

Glattstein et al.[14] examined the feasibility of applying adhesive lifters to the entrance bullet wound on human body surfaces to visualize the total nitrite patterns, as was reported for clothing and other exhibits above[12,13]. Figs. 5, 6, 7, and 8 demonstrate two cases in which it was impossible to observe visually/microscopically gunpowder residues around bullet entrance holes in the cadavers; however, total nitrite patterns were visualized on the adhesive lifters. In the case of a decomposed body (Fig. 5), a gunshot wound was found in the neck. Due to the blackish discoloration of the skin, the presence of soot or gunpowder tattooing could not be observed. However, the visualized total nitrite pattern of about 5 cm in diameter (Fig. 6) indicated a close-range shot. In the second case (Fig. 7), an entrance gunshot wound was found to the left parietal of the cadaver. No gunpowder particles were observed visually on hair before shaving, and no

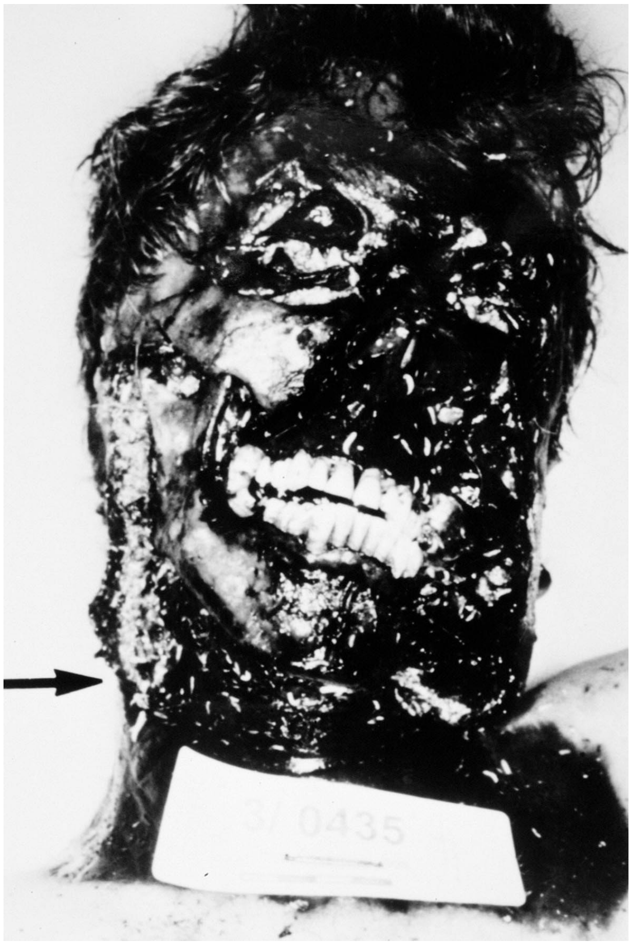

**FIGURE 5**. The head of a decomposed corpse; the arrow indicates the gunshot wound.

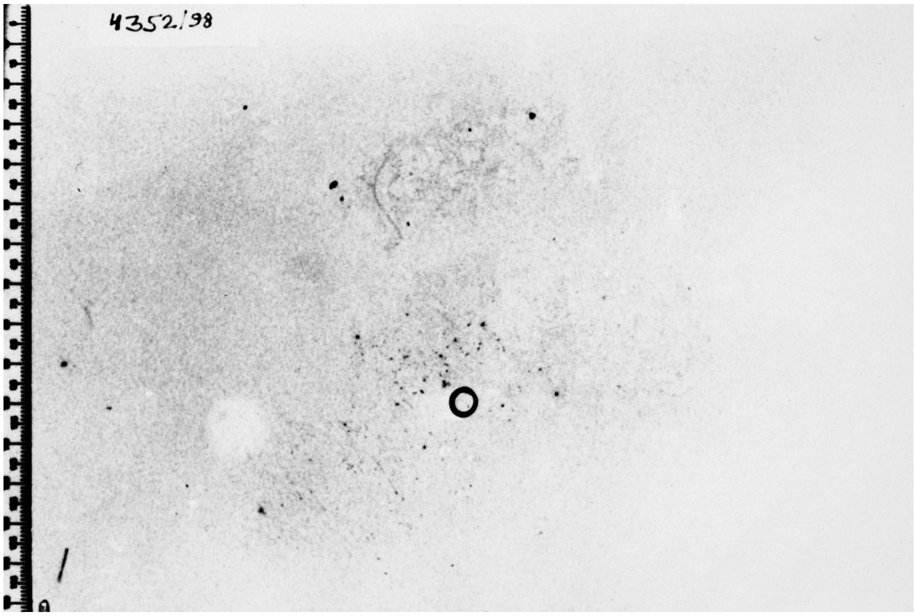

**FIGURE 6**. Black and white photograph of the visualized total nitrite pattern on the adhesive lifter applied to the gunshot wound of Fig. 5. The location of the bullet entrance hole is marked with a circle.

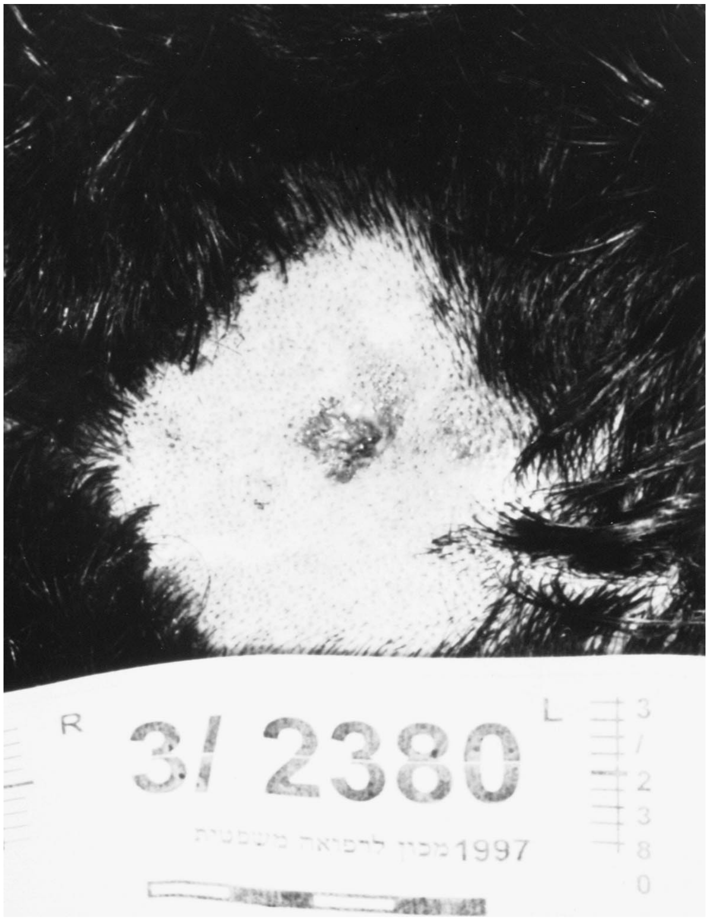

**FIGURE 7**. Gunshot wound at the head after shaving.

gunpowder tattooing was observed after shaving. The total nitrite pattern (Fig. 8) that was visualized on the adhesive lifter (that was applied before shaving) indicated a close-shot range. Stahling and Karlsson reported a similar method for lifting and visualizing gunpowder residues from skin[29].

## INSTRUMENTAL METHODS

X-ray fluorescence (XRF), atomic absorption spectroscopy (AAS), and neutron activation analysis (NAA) are the instrumental methods used by some laboratories to estimate the range of shooting[2].

In recent years, with the advent of the micro-XRF technology and its increasing use in forensic science for the elemental analysis of trace evidence, some of the laboratories examined its feasibility for shooting distance estimation[30,31]. Flynn et al.[30] evaluated the technique (Kevex Omicron micro-XRF) for the elemental analysis of GSR. They found that micro-XRF can detect GSR particles on the target substrate if the shooting distance is less than 30 cm. At greater distances, they could not detect GSR particles by this technique around the bullet wipe. Charpentier and Desrochers[31] used a similar instrument to analyze GSR from lead-free ammunition in which the lead in the primer was replaced with strontium and the bullet was plated

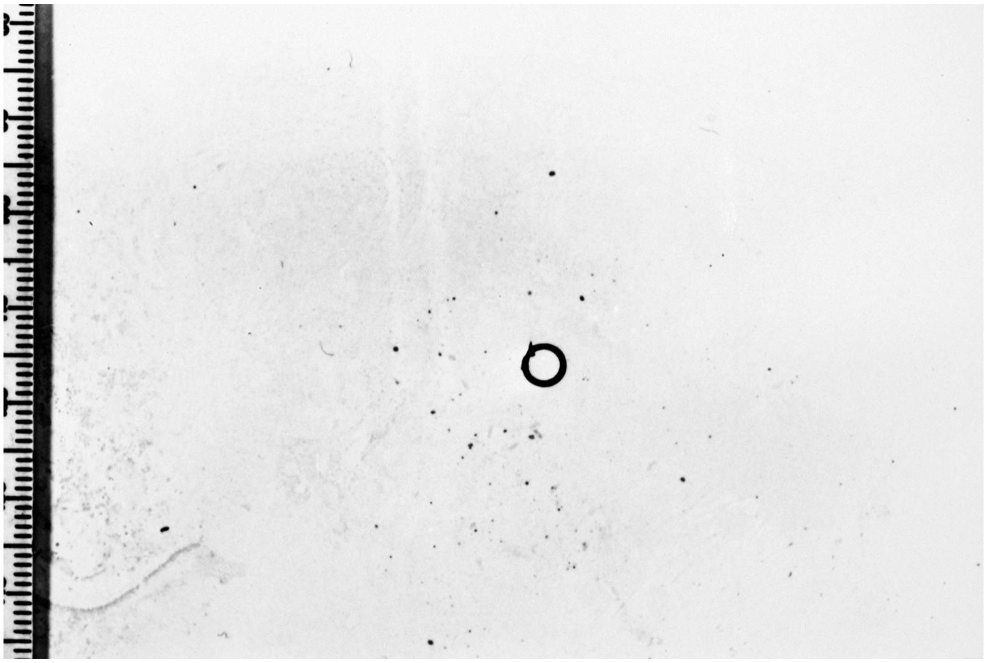

**FIGURE 8**. Black and white photograph of the visualized total nitrite pattern on the adhesive lifter applied on the hairy area of the wound (before shaving) in the Fig. 7. The location of the bullet entrance hole is marked with a circle.

with copper (TMJ). They found that the method allows the detection and quantification of strontium residues on the target up to a distance of 45 cm.

Brown et al.[32,33] studied the feasibility of an automated image analysis (IA) technique for shooting distance estimation. In the first study[32], they developed an IA procedure to measure the amount (number and area) of GSR particles around a gunshot wound. Measurements of GSR from test firings into goat hide were enhanced by using Alizarin Red S to stain the barium and lead components. A comparison was made between the amount of GSR detected on the stained skin sections and backscatter electron micrographs of the same sections. No significant differences were found between the two. Preliminary results indicated that there was a nonlinear, decreasing relationship between firing range and the amount of deposited GSR and that there was significant variation in the amount of GSR from shot to shot for firing ranges up to 20 cm. The second study[33] using the IA method was conducted on pig skin with a Ruger .22 semiautomatic rifle with CCI solid point for shooting ranges between contact and 45 cm.

## CONCLUSION

Recent developments in the methods of shooting distance estimation were primarily concentrated on the proposed protocols for combining color tests for metal deposits (mostly lead and copper) and for gunpowder residues around the bullet entrance holes. Lifting of the gunpowder residues by an adhesive lifter from the targets and applying the color test (Griess test or its modifications) on the lifter improves the methodology for clothing and introduces a new methodology for powder patterns on the human body and on exhibits that cannot be processed in the laboratory. Applying alkaline hydrolysis of the gunpowder residues before the color test may improve the results dramatically for some brands of ammunition. Specific color tests and the application of micro-XRF technique may assist in estimating shooting distances when lead-free ammunition is used. Fresh pig skin was found to be the most accurate simulant for human skin with regards to visual/microscopic examinations of the GSR residues.

In spite of those developments, some major inherent problems remain unsolved. Among these are, first, the maximum shooting distance that may be estimated is limited by the maximum range of GSR particles reaching the target and, second, the large variation in the amount of GSR from shot to shot is reflected in poor precision of the methodology. There is also a need to continue research on the assessment of the most accurate simulant materials for human skin with regards to all possible tests for shooting distance estimation.

## ACKNOWLEDGMENT

The authors would like to express their gratitude to A. Chaikovsky for his photography assistance.
